# Tackling global health security by building an academic community for One Health action

**DOI:** 10.1186/s40249-023-01124-w

**Published:** 2023-08-03

**Authors:** Xiao-Xi Zhang, Xin-Chen Li, Qi-Yu Zhang, Jing-Shu Liu, Le-Fei Han, Zohar Lederman, Janna M. Schurer, Patrícia Poeta, Md. Tanvir Rahman, Shi-Zhu Li, Kokouvi Kassegne, Kun Yin, Yong-Zhang Zhu, Shang Xia, Lu He, Qin-Qin Hu, Le-Shan Xiu, Jing-Bo Xue, Han-Qing Zhao, Xi-Han Wang, Logan Wu, Xiao-Kui Guo, Zhao-Jun Wang, Bernhard Schwartländer, Ming-Hui Ren, Xiao-Nong Zhou

**Affiliations:** 1 School of Global Health, Chinese Center for Tropical Diseases Research, Shanghai Jiao Tong University School of Medicine, Shanghai, People’s Republic of China; 2 Institute of One Health, Shanghai Jiao Tong University, Shanghai, People’s Republic of China; 3 Medical Ethics and Humanities Unit, Hong Kong University, Hong Kong, People’s Republic of China; 4 Center for One Health, University of Global Health Equity, Butaro, Rwanda; 5 Microbiology and Antibiotic Resistance Team, Department of Veterinary Sciences, University of Trás-os-Montes and Alto Douro, Vila Real, Portugal; 6 Associate Laboratory for Green Chemistry, Chemistry Department, University Nova of Lisbon, Lisbon, Portugal; 7 Veterinary and Animal Research Centre, University of Trás-os-Montes and Alto Douro, Vila Real, Portugal; 8 Associate Laboratory for Animal and Veterinary Sciences, University of Trás-os-Montes and Alto Douro, Vila Real, Portugal; 9 Department of Microbiology and Hygiene, Bangladesh Agricultural University, Mymensingh, Bangladesh; 10 National Institute of Parasitic Diseases at Chinese Center for Disease Control and Prevention (Chinese Center for Tropical Diseases Research), NHC Key Laboratory of Parasite and Vector Biology, WHO Collaborating Centre for Tropical Diseases, Shanghai, People’s Republic of China; 11 Walter and Eliza Hall Institute, Parkville, Australia; 12 Department of Medical Biology, University of Melbourne, Melbourne, Australia; 13 German Ministry of Foreign Affairs (Former Assistant Director General and Chef de Cabinet of Dr Tedros at the World Health Organization), Berlin, Germany; 14 School of Public Health, Peking University, Beijing, People’s Republic of China

**Keywords:** One Health, Global health, Academic community

## Abstract

**Background:**

One Health approach is crucial to tackling complex global public health threats at the interface of humans, animals, and the environment. As outlined in the One Health Joint Plan of Action, the international One Health community includes stakeholders from different sectors. Supported by the Bill & Melinda Gates Foundation, an academic community for One Health action has been proposed with the aim of promoting the understanding and real-world implementation of One Health approach and contribution towards the Sustainable Development Goals for a healthy planet.

**Main text:**

The proposed academic community would contribute to generating high-quality scientific evidence, distilling local experiences as well as fostering an interconnected One Health culture and mindset, among various stakeholders on different levels and in all sectors. The major scope of the community covers One Health governance, zoonotic diseases, food security, antimicrobial resistance, and climate change along with the research agenda to be developed. The academic community will be supported by two committees, including a strategic consultancy committee and a scientific steering committee, composed of influential scientists selected from the One Health information database. A workplan containing activities under six objectives is proposed to provide research support, strengthen local capacity, and enhance global participation.

**Conclusions:**

The proposed academic community for One Health action is a crucial step towards enhancing communication, coordination, collaboration, and capacity building for the implementation of One Health. By bringing eminent global experts together, the academic community possesses the potential to generate scientific evidence and provide advice to local governments and international organizations, enabling the pursuit of common goals, collaborative policies, and solutions to misaligned interests.

**Graphical Abstract:**

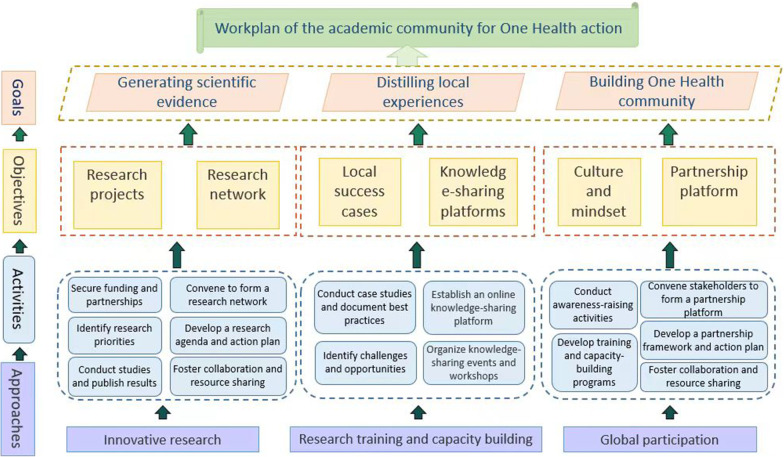

**Supplementary Information:**

The online version contains supplementary material available at 10.1186/s40249-023-01124-w.

## Background

The One Health approach, centered around holistic thinking at the interface of humans, animal, and the environment, aims to promote the health and sustainable development of these sectors through multi-sectoral, interdisciplinary, and cross-border collaboration [1]. It is an evolved way of thinking for the international community as it responds to global public health threats, with developmental milestones reached over the past few years.

In October 2022, the Food and Agriculture Organization of the United Nations (FAO), the United Nations Environment Programme (UNEP), the World Health Organization (WHO), and the World Organization for Animal Health (WOAH) jointly released the One Health Joint Plan of Action (OHJPA) [2], marking renewed attention to global action with a One Health theme. However, the determinants of successful One Health approaches that can be adapted to local contexts, are still unclear. There is a need for more empirical evidence from global, regional, and national levels, to enhance the overall benefit of human, animal, and environmental health, as well as to undertake research priority setting to support the quadripartite OHJPA.

Supported by Bill & Melinda Gates Foundation, an academic community for One Health action has been proposed by Shanghai Jiao Tong University in Shanghai China, with the aim of promoting the understanding, contribution and real-world implementation of the One Health approach towards the Sustainable Development Goals (SDGs). In order to strategize the construction of this academic community, a Scientific Steering Committee (SSC) was jointly formed up with a wide engagement of more than 26 experts and institutions over 18 countries and regions (Additional file 1), who have been identified, reached and invited using the One Health Information database [3].

Based on the in-depth exchanges and consensus of SSC, the academic community will commit to providing robust scientific evidence, enabling academic contributions in One Health development, addressing technical development gaps, and synthesizing empirical experiences in One Health practice to tailor strategies for specific social-ecological settings. This article debriefs the proposed research agenda for the proposed academic community for One Health action in the initiate stage, including overarching goal, tasks, workplan followed by research plans.

## Overarching goal

The vision of the academic community for One Health action is to build a stronger international community to promote One Health approach that aligns with the SDGs. To achieve this vision, the academic community aims to develop the understanding of the determinants and functions of successful One Health practice in order to improve the effectiveness of One Health implementation.

The academic community can contribute to achieving the goal by:

(i) Providing high-quality scientific evidence through collaborative research, joint publications, and conferences on issues such as (re-)emerging infectious diseases, climate change, and antimicrobial resistance (AMR).
(ii) Distilling local experiences and analyzing current One Health practices to prioritize national strategies and international collaborations.
(iii) Fostering an interconnected One Health community, where partners have visibility of resources and policymakers have access to One Health solutions.

## Tasks

The academic community will develop a research agenda for global partners working in One Health and promote information sharing by providing a collaborative body for members. The academic community will also connect different disciplines and stakeholders at national, regional, and global levels to promote innovation in implementation and increase the impact of One Health work, including through cooperation with international organizations such as the WHO and the World Bank.

The academic community also serves as an international exchange and cooperation platform for One Health solutions tailored for the diverse socio-ecological settings within the networks, which also provides an additional benefit to the inter-governmental collaborations that extend beyond the realm of One Health.

Finally, the academic community will advocate for awareness of the links between human, animal, and environmental health and engage with policy makers at both national and international levels to promote the inclusion of One Health measures. The academic community will also collaborate with non-governmental organizations, academic institutions, and others in the public and private sectors to promote education and public awareness of One Health.

## Workplan

The organizing structure of the academic committee has been proposed. A Strategic Consultancy Committee (SCC) and a Scientific Steering Committee (SSC) will be constructed to form the leadership of the academic community. The SCC, composed of high-level key opinion leaders, is responsible for developing strategic plans and action guides for the academic committee, supervising the work of SSC and secretariat and leveraging resources to catalyze the implementation of workplans. The SSC is composed of top-ranking academic experts, who have been selected from the One Health information database [3] based on the individual academic contribution to One Health evaluated by publication citation, impact, type, and author contribution. It is responsible for developing detailed annual workplans for the academic committee, and providing technical guidance for research projects, and leading world-class academic progress in One Health. The secretariat will be located at Shanghai Jiao Tong University and oversee the routine operation and management of the academic community.

**Fig. 1:**
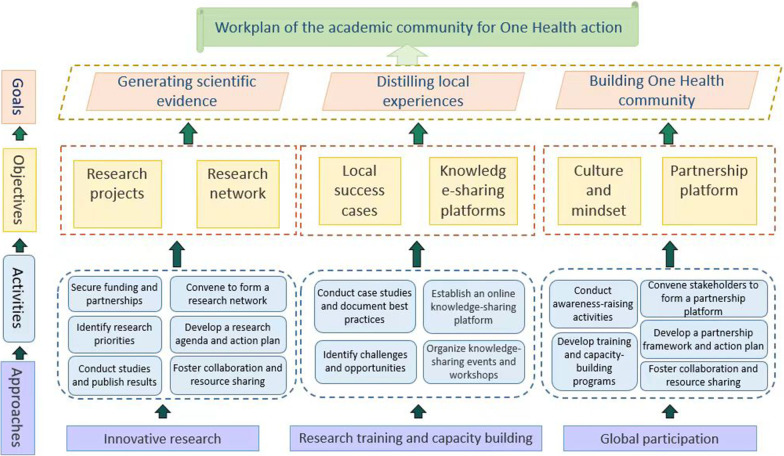
Flowchart of the academic community’s workplan

Under the three goals of the academic community, six objectives have been developed: conducting collaborative research project (objective 1), expanding interconnected research network (objective 2), identifying and disseminating local cases (objective 3), promoting knowledge- and data- sharing platform (objective 4), fostering One Health culture and mindset (objective 5), and engaging broader partnership (objective 6). The workplan to achieve these objectives and goals is shown in Fig. 1, with each objective being pursued through a series of activities and supported by overarching approaches.

## Research agenda

Based on overarching goals of the academic community for One Health action, research priorities to support the community will focus following five areas, including One Health governance, control of zoonotic infections, food security and food safety, control of AMR, and impact evaluation of climate changes on health.

### One Health governance

One Health governance refers to the development, implementation, and oversight of policies that support the One Health approach. It encompasses eight dimensions, including public participation, rule of law, transparency, responsiveness, consensus orientation, fairness and inclusivity, effectiveness and efficiency, and policy support [4]. An effective governance framework is crucial for countries to detect, mitigate, and prevent public health threats [4].

A major challenge is the lack of coordination between different sectors and stakeholders [5]. Many countries have siloed approaches to health, agriculture, and the environment, which hinder the adoption of the One Health ethos. Additionally, the lack of a shared understanding of the roles and responsibilities of different actors in One Health, as well as insufficient human and financial commitments, exacerbate this challenge.

To address this issue, the academic community should facilitate the development of effective One Health governance frameworks that span multiple sectors and disciplines, drawing from lessons learned towards best practices. The academic community will also advocate for the establishment of a global governance framework for One Health, providing an international reference point for countries aiming to improve their own One Health governance.

### Control of zoonotic infections

Zoonotic diseases are a high-priority target for the One Health approach. Zoonotic diseases are challenging for global surveillance, prevention, and control measures. One of the main factors is the emergence of these diseases, which highlights the need for a better understanding of the dynamics of pathogen spillover and transmission between animals and humans so that intervention strategies could be developed to zoonoses more effectively [6]. Additionally, there is a lack of understanding regarding the drivers of zoonotic disease emergence, including changes in climate, land use, and wildlife trade [7].

To address these challenges, the academic community will facilitate research to identify the drivers of zoonotic disease emergence and enhance surveillance and prevention strategies. This will require interdisciplinary collaboration among public health, veterinary medicine, wildlife ecology, and the social and economic sciences. The academic community will also strengthen countries’ capacities to detect, report, and respond to zoonotic disease outbreaks by three activities: (i) expanding laboratory capacity, including improving laboratory professional level, building reference laboratory networks, standardizing standard operating procedures, etc.; (ii) improving disease surveillance efficiency, including applying innovative surveillance tools, enhancing surveillance and response systems, improving the surveillance evaluation approaches, etc.; and (iii) strengthening animal health systems, including enlarging animal surveillance networks, developing animal disease alarming system, optimizing animal health management, etc.

### Food security and food safety

Food security and food safety is a complex issue that affects all aspects of human life. It involves ensuring that people have access to sufficient, safe, and nutritious food that is appropriate for their culture and lifestyle [8]. One of the key objectives of the academic community is to improve global food security and food safety through the One Health approach.

Currently, over 820 million people worldwide suffer from chronic hunger, while an additional 2 billion people suffer from malnutrition due to a lack of essential micronutrients in their diets [9]. Climate change further exacerbates this situation by causing more frequent and severe weather events that reduce agricultural productivity and accessibility. Additionally, the world’s population is expected to reach 9.7 billion by 2050, which will increase the demand for food by 70% [10]. These challenges highlight the need for the One Health approach to address food insecurity and malnutrition.

To improve food security, the academic community will promote sustainable agricultural practices, enhance the resilience of supply chains to climate change and other shocks, reduce food waste, improving food safety, and enhance food accessibility. The One Health approach will address these objectives by working with organizations and governments to incorporate the interdependence of human, animal, and environmental health in their initiatives.

### Control of antimicrobial resistance

AMR is a major public health threat and another key focus of the academic community. AMR occurs when bacteria, viruses, fungi, or parasites develop resistance to antimicrobial drugs, making it more difficult to treat infections [11]. The misuse of antimicrobials in healthcare, agriculture, and animal husbandry is a significant driver of AMR, but effective surveillance can slow its spread.

One of the main challenges in addressing AMR is the lack of awareness and understanding among the public who access drugs over the counter, and healthcare professionals who administer them in bulk, both behaviors that contribute to resistance. Additionally, developing new antimicrobials is slow and costly, and even newly developed drugs can quickly become ineffective if they are similarly misused [12].

To combat AMR, the academic community targets at educating the public and healthcare professionals about the risks of AMR and the importance of appropriate antimicrobial use, as well as improving access to diagnostic tests to ensure that antimicrobials are only used when necessary.

In addition to these efforts, the academic community should work to improve surveillance and monitoring of AMR in various One Health components including wild life and migratory birds along with development of better methods for tracking the spread of resistant pathogens between animals, humans and ecosystems. The academic community also supports the development of rapid and cost-effective point-of-care testing AMR diagnostic technologies, alternative treatments, and vaccines to mitigate the impact of AMR. Through these efforts, the academic community hopes to slow the spread of AMR and preserve the effectiveness of crucial drugs.

### Impact evaluation of climate changes on health

Climate change is one of the most pressing global challenges of our time. Its impacts are vast and far-reaching, affecting not only the natural world but also human health and well-being. Disadvantaged and vulnerable populations are disproportionately affected. Climate change exacerbates existing health problems and poses new risks, such as increased frequency and severity of extreme weather events, changing patterns of infectious diseases, and food and water insecurity [13]. Addressing the impacts of climate change requires a multidisciplinary and multisectoral approach, with a focus on reducing greenhouse gas emissions, adapting to the changing climate, and promoting sustainable development.

The One Health approach provides an essential framework for addressing the health impacts of climate change [14]. Key strategies include promoting sustainable agriculture and food systems, improving water and sanitation infrastructure, developing early warning systems for extreme weather events and infectious diseases, etc. In addition, the One Health approach emphasizes the importance of collaboration and engagement with all stakeholders, including governments, civil society, the private sector, and local communities, to build resilience and promote sustainable development in the face of climate change. The academic community will also support the implementation of sustainable, low-emission, resilient practices in industries such as agriculture, transportation, and energy to mitigate the impacts of climate change.

## Recommendations

To build an academic community for One Health action, four approaches have been proposed as follows.

### Resources connection

The academic community connects resources to support interdisciplinary research in the field of One Health. This includes advocating funding agency to put One Health on their high profile and linking funding opportunities to research projects that investigate the links between human, animal, and environmental health. Meanwhile, the academic community supports joint research programs and collaborations across different sectors and countries and also cultivate the research competitiveness for professionals in One Health to improve their capabilities to obtain funding and resources.

### Policy advocacy

The academic community serves as a bridge to translate scientific knowledge from research to evidence-based solutions for policy makers. Through applying both bottom-up and top-down approaches, the academic community supports implementation science to identify local needs and real gaps, and builds up dialogues between local practitioners, scientists, funders and policy makers. Meanwhile, the academic community can also contribute to developing innovative mechanisms to connect the local with the regional, and to scale up regional experiences to global policy agenda.

### Capacity building

The academic community supports training programs and other capacity-building initiatives to strengthen the research capabilities of individuals and organizations working in One Health fields. This includes training in research paradigms, methods, experimental practices, and data analysis, as well as the development of One Health related disciplines and research networks, with an emphasis on early career researchers and researchers from low- and middle-income countries.

### Global engagement

The academic community develops communication and engagement strategies to promote public awareness of One Health, and encourages diversity of the participants in One Health initiatives. It also coordinates stakeholders from around the world, together with representatives from the governments, non-governmental organizations, academic institutions, and the private sector, to promote One Health policies and practices.

## Conclusions

This article proposes to foster an academic community for One Health action, which is expected to incorporate the experience of a diverse variety of experts and stakeholders, who will be organized under the structure of SCC and SSC. Overarching research agenda followed by specific workplans and activities has been elaborated in this article, based on the exchanges and consensus of SSC. Multifaceted approach, that involves systematic thinking, evidence-based activities, transparent mechanisms, education, and communication, should be adapted profoundly in building up the community.

With the tasks of promoting wider and deeper collaboration in research and practice, the community will contribute to producing novel scientific evidence, facilitating dialogues among different stakeholders, and advising for national and international policy, in its efforts to tackle global public health security at the human-animal-environment interface.

## Supplementary Information

**Additional file 1: Table S1.** The list of geographic distributions and research areas of Scientific Steering Committee (SSC) members of the proposed academic community

## Data Availability

The full study protocol and the datasets, are available, following manuscript publication, upon request from the corresponding author.

## Abbreviations

AMR: Antimicrobial resistance
COVID-19: Coronavirus disease 2019
FAO: Food and Agriculture Organization
OHJPA: One Health Joint Plan of Action
SCC: Strategic Consultancy Committee
SDGs: Sustainable Development Goals
SSC: Scientific Steering Committee
UNEP: United Nations Environment Programme
WHO: World Health Organization
WOAH: World Organization for Animal Health
