# Supplementary material for: Tackling global health security by building an academic community for One Health action
Source: Infect Dis Poverty. 2023 Aug 3;12:70. doi: 10.1186/s40249-023-01124-w (PMC10398903; doi:10.1186/s40249-023-01124-w)
Supplement: Supplementary file 1 — Additional file 1: Table S1. The list of geographic distributions and research areas of Scientific Steering Committee (SSC) members of the proposed academic community [file 40249_2023_1124_MOESM1_ESM.docx]

**Additional file 1**

Table S1 The list of geographic distributions and research areas of Scientific Steering Committee (SSC) members of the proposed academic community

| **Country** | **Number** | **Research Areas** |
| --- | --- | --- |
| UK | 2 | AMR, epidemiology |
| Australia | 1 | Zoonoses |
| Bangladesh | 2 | Zoonoses |
| Belgium | 1 | Veterinary health |
| Brazil | 1 | Immunology |
| Canada | 2 | One Health, ecosystem |
| Ecuador | 1 | Epidemiology |
| India | 1 | Veterinary health |
| Israel | 1 | Clinical medicine |
| Italy | 1 | Vector-borne diseases |
| Kenya | 1 | Climate change, food security |
| Nepal | 1 | Wildlife |
| Norway | 1 | Life science |
| Portugal | 1 | AMR |
| Rwanda | 1 | Global health governance |
| Thailand | 1 | Ecohealth |
| Switzerland | 1 | One Health |
| China | 6 | Zoonoses, AMR, climate change, food security, governance |

Note: This list is based on the initial discussion of the proposed academic community, which is in the stage of expansion and updates.
